# Complete genome sequences of *Francisella noatunensis* subsp*. orientalis* strains FNO12, FNO24 and FNO190: a fish pathogen with genomic clonal behavior

**DOI:** 10.1186/s40793-016-0151-0

**Published:** 2016-04-12

**Authors:** Lucas Amorim Gonçalves, Siomar de Castro Soares, Felipe Luiz Pereira, Fernanda Alves Dorella, Alex Fiorini de Carvalho, Gabriel Magno de Freitas Almeida, Carlos Augusto Gomes Leal, Vasco Azevedo, Henrique César Pereira Figueiredo

**Affiliations:** National Reference Laboratory for Aquatic Animal Diseases (AQUACEN), Ministry of Fisheries and Aquaculture, Federal University of Minas Gerais, Belo Horizonte, Brazil; Laboratory of Cellular and Molecular Genetics (LGCM), Federal University of Minas Gerais, Belo Horizonte, Brazil

**Keywords:** Complete genome sequencing, Fish pathogen, Genetic clonal behavior

## Abstract

The genus *Francisella* is composed of Gram-negative, pleomorphic, strictly aerobic and non-motile bacteria, which are capable of infecting a variety of terrestrial and aquatic animals, among which *Francisella noatunensis* subsp. *orientalis* stands out as the causative agent of pyogranulomatous and granulomatous infections in fish. Accordingly, *F. noatunensis* subsp*. orientalis* is responsible for high mortality rates in freshwater fish, especially Nile Tilapia. In the current study, we present the genome sequences of *F. noatunensis* subsp. *orientalis* strains FNO12, FNO24 and FNO190. The genomes include one circular chromosome of 1,859,720 bp, consisting of 32 % GC content, 1538 coded proteins and 363 pseudogenes for FNO12; one circular chromosome of 1,862,322 bp, consisting of 32 % GC content, 1537 coded proteins and 365 pseudogenes for FNO24; and one circular chromosome of 1,859,595 bp, consisting of 32 % GC content, 1539 coded proteins and 362 pseudogenes for FNO190. All genomes have similar genetic content, implicating a clonal-like behavior for this species.

**Electronic supplementary material:**

The online version of this article (doi:10.1186/s40793-016-0151-0) contains supplementary material, which is available to authorized users.

## Introduction

In 1922, Edward Francis (1872–1957), an American bacteriologist, described the bacterium that causes tularemia in humans, *Francisella tularensis*. This bacterium is the most studied of its genus [1, 2]. Until recently, the genus *Francisella* consisted of only two species: *F. tularensis* and *F. philomiragia*; however, new species and new strains were isolated, such as *F. noatunensis* and the subspecies *F. noatunensis* subsp. *orientalis* [1], the latter being recognized as one of the most important pathogens of cultured tilapia (*Oreochromis**spp*.) [3].

*F. noatunensis* subsp. *orientalis* is the etiologic agent of pyogranulomatous and granulomatous infections in fish. In the last few years, *F. noatunensis* subsp. *orientalis*has been responsible for a large number of deaths of tilapia and other freshwater species cultured in the United States, the United Kingdom, Japan, Taiwan, Jamaica, Costa Rica, Brazil and some other Latin American regions [4–6]. Nevertheless, besides infecting important cultivable species such as tilapia, threeline grunt (*Parapristipoma trilineatum*) and hybrid striped bass (*Morone chrysops* X *Morone saxatilis*), this bacterium is also capable of infecting wild fish such as guapote tigre (*Parachromis managuensis*) [4, 5].

Although the disease caused by this species presents with a high mortality rate during outbreaks and has been reported in several countries, the phylogenomic relationships among isolates from different countries and the evolutionary history of this pathogen are still poorly characterized. Therefore, the strains presented herein were isolated from three different regions and outbreaks to characterize the genetic diversity of the microorganism *F. noatunensis* subsp. *orientalis* strains FNO12, FNO24 and FNO190.

## Organism information

### Classification and features

**Fig. 1:**
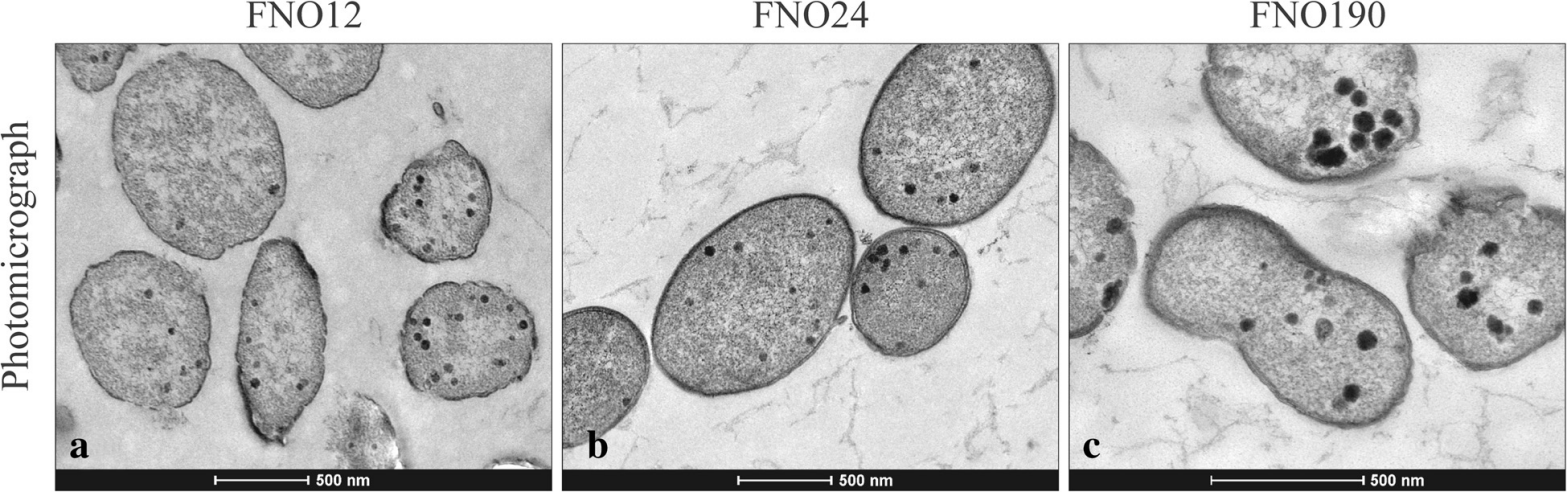
Photomicrograph of the F. noatunensis subsp. orientalis strains. The strains FNO12, FNO24 and FNO190 are represented, respectively, by sections **a**, **b** and **c**

**Fig. 2:**
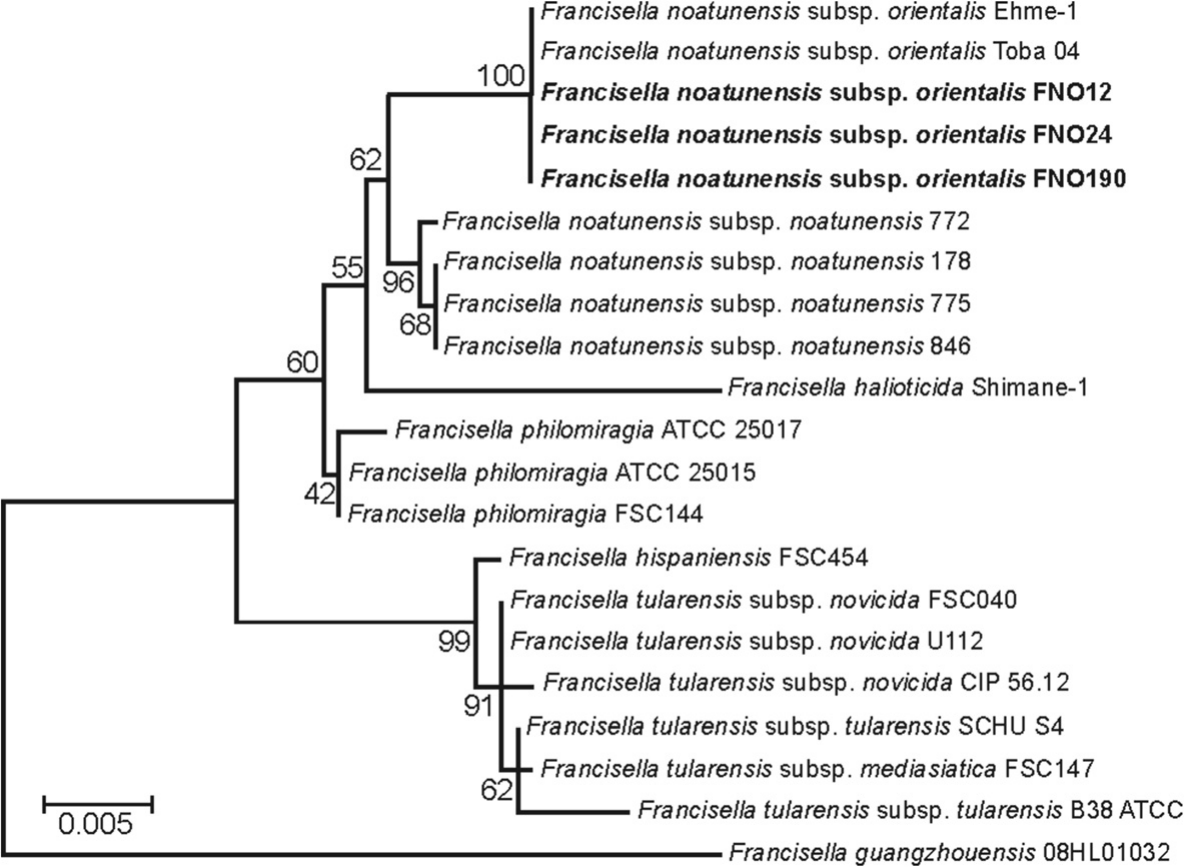
Phylogenetic tree of the F. noatunensis subsp. orientalis strains. Phylogenetic tree of the F. noatunensis subsp. orientalis strains FNO12, FNO24 and FNO190 representing their relative position in the genus Francisella based on 16S sequences. The statistical method used was maximum likelihood, and the bootstrap number was 1000. Thus, the values next to the nodes represent the percentage of the number of times, in 1000 repetitions, in which that clade was formed

This *Francisella* genus, from phylum *Proteobacteria*, class *Gammaproteobacteria*, order *Thiotrichales*, and family *Francisellaceae*, is a strictly aerobic, non-motile, pleomorphic, and Gram-negative bacteria of 0.5–1.5 μm (Table 1 and Fig. 1). It is negative for nitrate reduction as well as adonitol, arabinose, cellobiose, esculin, galacturonate, glucuronate, malonate, mannitol, melibiose, raffinose, rhamnose, palatinose, and 5-ketogluconate fermentation. In contrast, it has C14 lipase, cystine arylamidase, para-phenylalanine deaminase, tetrathionate reductase, trypsin, urease, valine arylamidase, α-chymotrypsin, α-fucosidase, α-galactosidase, α-mannosidase, and β-glucuronidase activity, as well as acid production from lactose. Additionally, it is positive for acid phosphatase, alkaline phosphatase, C4 and C8 esterase, lipase, naphtol-AS-BI-phosphohydrolase, β-lactamase activity, and acid production from maltose [7]. Using the 16S RNA sequences with 1516 bp of FNO12, FNO24, and FNO190 with the neighbor-joining method based on 1000 randomly selected bootstrap replicates of alignments using Mega6 software [8], a phylogenetic tree showing these strains positioned in a species-specific clade was constructed (Fig. 2).

**Table 1:** Classification and general features of *Francisella noatunensis* subsp. *orientalis* strains FNO12, FNO24 and FNO190 according to the MIGS recommendations [9]

MIGS ID	Property	Term	Evidence code^a^
	Classification	Domain *Bacteria*	TAS [26]
		Phylum *Proteobacteria*	TAS [27]
		Class *Gammaproteobacteria*	TAS [28]
		Order *Thiotrichales*	TAS [29]
		Family *Francisellaceae*	TAS [30]
		Genus *Francisella*	TAS [31, 32]
		Species *Francisella noatunensis* subsp. *orientalis*	TAS [33]
		Type strain FNO12, FNO24 and FNO190	IDA
	Gram stain	Gram-negative	TAS [33]
	Cell shape	pleomorphic	TAS [33]
	Motility	Non-motile	TAS [33]
	Sporulation	Not reported	NAS
	Temperature range	Mesophilic (15–34 °C)	TAS [33]
	Optimum temperature	<25 °C	TAS [33]
	pH range; Optimum	Not reported	NAS
	Carbon source	Not reported	NAS
MIGS-6	Habitat	FNO12 – Nile tilapia kidney	NAS
		FNO24 – Nile tilapia spleen	
		FNO190 – Nile tilapia spleen	
MIGS-6.3	Salinity	Not reported	NAS
MIGS-22	Oxygen requirement	Strictly aerobic	TAS [33]
MIGS-15	Biotic relationship	Intracellular facultative pathogen	TAS [7]
MIGS-14	Pathogenicity	Pathogenic for fish	TAS [7]
MIGS-4	Geographic location	FNO12 – Brazil/State of Minas Gerais/Areado city	NAS
		FNO24 – Brazil/State of Minas Gerais/Alterosa city	
		FNO190 – Brazil/State of São Paulo/Santa fé do Sul city	
MIGS-5	Sample collection	FNO12– Mai 5, 2012	NAS
		FNO24 – Mai 5, 2012	
		FNO190 – Nov 10, 2013	
MIGS-4.1	Latitude	FNO12 – 21° 21′ S	NAS
		FNO24 – 21° 14′ S	
		FNO190 – 20° 12′ S	
MIGS-4.2	Longitude	FNO12 – 46° 08′ W	NAS
		FNO24 – 46° 08′ W	
		FNO190 – 50° 55′ W	
MIGS-4.4	Altitude	FNO12 – ~1,006	NAS
		FNO24 – ~848	
		FNO190 – 370	

^a^Evidence codes - *IDA* Inferred from Direct Assay, *TAS* Traceable Author Statement (i.e., a direct report exists in the literature), *NAS*, Non-traceable Author Statement (i.e., not directly observed for the living, isolated sample, but based on a generally accepted property for the species, or an anecdotal evidence). These evidence codes are from the Gene Ontology project [11]

## Genome sequencing information

### Genome project history

In the present study, the nucleotide sequence of the *F. noatunensis* subsp. *orientalis* FNO12, FNO24 and FNO190 complete genomes was determined. Sequencing and assembly were performed by the National Reference Laboratory for Aquatic Animal Diseases, and annotation was performed by the Laboratory of Cellular and Molecular Genetics. Both laboratories are located at the Federal University of Minas Gerais, Belo Horizonte, Minas Gerais, Brazil. Source DNA of these three strains are available at culture collection of AQUACEN. Table 2 presents the project information and its association with MIGS version 2.0 compliance [9].

**Table 2:** Project information

MIGS ID	Property	Term/Strains		
		FNO12	FNO24	FNO190
MIGS-31	Finishing quality	Finished	Finished	Finished
MIGS-28	Libraries used	Fragment	Fragment	Fragment
MIGS-29	Sequencing platforms	Illumina MiSEQ	Ion Torrent PGM™	Ion Torrent PGM™
MIGS-31.2	Fold coverage	1382.15	79.82	203.43
MIGS-30	Assemblers	Edena	Mira and Newbler	Mira and Newbler
MIGS-32	Gene calling method	RAST	RAST	RAST
	Locus Tag	FNO12	FNO24	FNO190
	Genbank ID	CP011921	CP011922	CP011923
	Genome Database release	2015/6/20	2015/6/20	2015/6/20
	GOLD ID	Gb0109929	Gb0109930	Gb0109931
	BIOPROJECT	PRJNA232116	PRJNA234502	PRJNA240882
MIGS-13	Source Material Identifier	FNO12	FNO24	FNO190
	Project relevance	Fish pathogen associated with a large number of deaths of tilapia and other freshwater species	Fish pathogen associated with a large number of deaths of tilapia and other freshwater species	Fish pathogen associated with a large number of deaths of tilapia and other freshwater species

### Growth conditions and genomic DNA preparation

*F. noatunensis* subsp. *orientalis* strains FNO12, FNO24 and FNO190 were isolated from three different outbreaks from Nile tilapia fish farms. Swabs of kidney (FNO12) and spleen (FNO24 and FNO190) tissues from each fish were sampled aseptically, streaked onto cysteine heart agar supplemented with 2 % bovine hemoglobin (BD Biosciences, USA) and incubated at 28 °C for 4–7 days [7]. The isolates were stored at -80 °C in Mueller-Hinton cation-adjusted broth supplemented with 2 % VX supplement (Laborclin, Brazil), 0.1 % glucose, and 15 % glycerol. The isolates were thawed, streaked onto CHAH and incubated at 28 °C for 48–72 h. Genomic DNA was extracted by the use of the Maxwell 16® Research Instrument (Promega, USA) according to the manufacturer’s recommendations. Briefly, (i) 2 x 10^9^ cells were lysed in the presence of a chaotropic agent and a detergent, (ii) nucleic acids were bound to silica magnetic particles, (iii) bound particles were washed and isolated from other cell components, and (iv) nucleic acids were eluted into a formulation for sequencing. Genomic DNAs were measured using Qubit 2.0 Fluorometer (Life Technologies, Thermo Scientific, USA) and yield of DNA were 64.8 ng/μL (FNO12), 58.0 ng/μL (FNO24) and 54.4 ng/μL (FNO190). Purity of DNAs (UV A_260_/A_280_) was accessed by NanoDrop 2000 Spectrophotometer (Thermo Scientific, USA). Ratios for each sample were 1.89, 1.95, and 1.96 for FNO12, FNO24 and FNO190, respectively. The extracted DNA was stored at -80 °C until use.

### Genome sequencing and assembly

The genome sequencing of the FNO12 strain was performed with the MiSEQ platform (Illumina®, USA), while the genome sequencing of the FNO24 and FNO190 strains was performed with the Ion Torrent Personal Genome Machine™ (Life Technologies, USA). MiSEQ used the Nextera DNA Library Preparation Kit while PGM used the Ion PGM 200 bp Sequencing Kit. The quality of the raw data was analyzed using FastQC [10], and the assembly was performed using the Edena 2.9 [11], Mira 3.9 [12] and Newbler 2.9 (Roche, USA) as the applied *ab initio* strategy. The assemblies of FNO12, FNO24 and FNO190 produced a total of 15, 57 and 16 contigs, respectively. The first strain resulted in ~1382-fold, coverage, the second had a value of ~79-fold, coverage, and the third had a value of ~203-fold coverage,. Additionally, the strains FNO12, FNO24 and FNO190 presented an N50 value of 275,043 bp, 87,100 bp, and 237,022 bp, respectively. A super scaffold for FNO12 was produced with an optical map as a reference using restriction enzyme NheI, on MapSolver software (OpGen Technologies, USA). The remaining gaps were filled through the use of CLC Genomics Workbench 7 (Qiagen, USA) by mapping the raw data in gap flank repeated times until the overlap was found. For FNO24 and FNO190, the complete genome of FNO12 was used as a reference to construct the super scaffolds on CONTIGuator 2.0 software [13], and gap filling was conducted as described for strain FNO12. All the raw sequencing data were mapped onto the each final genome and the lack of contamination with other genomes were confirmed by the coverage and the low number of unmapped reads.

### Genome annotation

Automatic annotation was performed using the RAST software [14]; tRNA and rRNA predictions were conducted using the tRNAscan-SE Search Server [15] and the RNAmmer [16], respectively. Manual curation of the annotation was done using Artemis software [17] and the UniProt database [18]. All putative frameshifts were manually curated based on the raw data coverage in CLC Genomics Workbench 7 software (Qiagen, USA), which was used to correct indel errors in regions of homopolymers.

## Genome properties

**Fig. 3:**
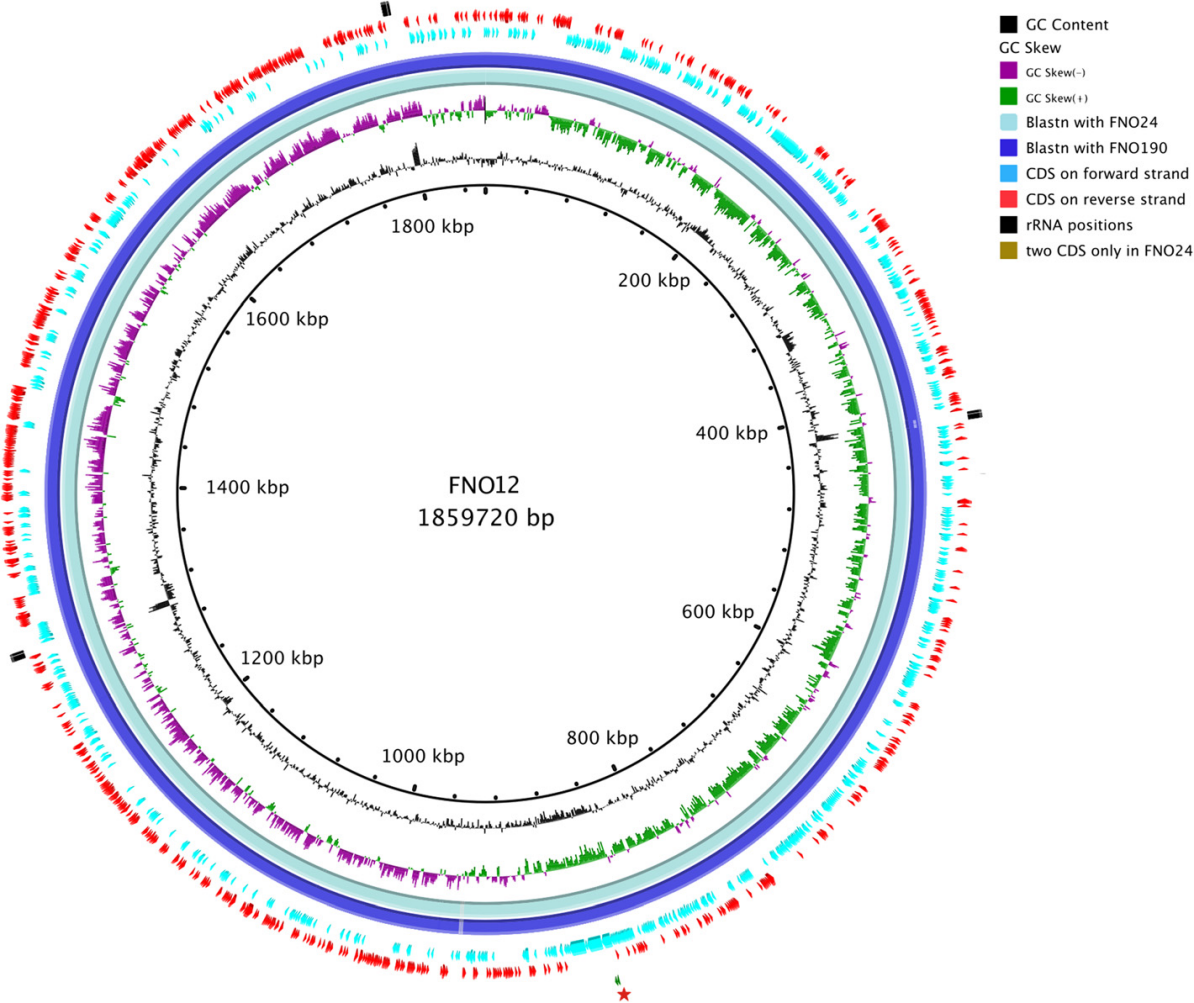
Graphical circular map of *F. noatunensis* subsp. *orientalis* strain FNO12 in comparison with FNO24 and FNO190 (presented in this work) and LADL-07-285A and Toba04 (deposited in GenBank). From outside to the center: two CDSs only present in FNO24 (close to red star), tRNA positions, rRNA positions, CDSs on reverse strand, CDSs on forward strand, BlastN hits with Toba04 strain, BlastN hits with LADL-07-285A strain, BlastN hits with FNO190, BlastN hits with FNO24, GC skew and GC content

The genomes are each comprised of a circular chromosome with sizes of 1,859,720 bp, 1,862,322 bp, and 1,859,595 bp for FNO12, FNO24, and FNO190, respectively (Table 3). The GC content in the three strains is 32 %, and the number of pseudogenes is relatively high (363 on average). Strain FNO24 had more protein coding genes, and one RNA-coding gene fewer than the other two strains. For the FNO12 and FNO190 strains, 1280 genes were annotated with functional prediction, whereas for strain FNO24, 1282 genes were annotated. Each genome contained 621 CDSs classified as hypothetical proteins by the COG database [19]. Table 4 summarizes the number of genes associated with general COG functional categories. Figure 3 shows the comparison of FNO12 with FNO24, FNO190 (presented in this study) with the other two strains deposited in GenBank (*F. noatunensis* subsp. *orientalis* strains LADL-07-285A and Toba04, accession numbers: CP006875 and CP003402, respectively).

**Table 3:** Genome statistics

Attribute	Strain					
	FNO12		FNO24		FNO190	
	Value	% of total^a^	Value	% of total^a^	Value	% of total^a^
Genome size (bp)	1,859,720	100.00	1,862,322	100.00	1,859,595	100.00
DNA coding (bp)	1,348,998	72.53	1,343,370	72.13	1,350,675	72.63
DNA G + C (bp)	600,797	32.30	601,431	32.29	600,768	32.30
DNA scaffolds	1	100.00	1	100.00	1	100.00
Total genes	1,951	100.00	1,952	100.00	1,951	100.00
Protein coding genes	1,538	78.83	1,537	78.73	1,539	78.78
RNA genes	50	2.56	49	2.51	50	2.56
Pseudo genes	363	18.60	365	18.62	362	18.55
Genes with function prediction	1,280	65.60	1,282	65.67	1,280	65.60
Genes assigned to COGs	1,327	68.01	1,327	67.98	1,326	67.96
Genes with Pfam domains	1,562	80.06	1,564	80.12	1,561	80.01
Genes with signal peptides	128	6.56	128	6.55	126	6.45
Genes with transmembrane helices	531	27.21	531	27.20	534	27.37
CRISPR repeats	0	0	0	0	0	0

^a^The total is based on either the size of the genome in base pairs or the total genes in the annotated genome

**Table 4:** Number of genes associated with general COG functional categories

Code	Strains						Description
	FNO12		FNO24		FNO190		
	Value	% age	Value	% age	Value	% age	
J	152	8.00	152	7.99	152	8.00	Translation, ribosomal structure and biogenesis
A	1	0.05	1	0.05	1	0.05	RNA processing and modification
K	47	2.47	47	2.47	47	2.47	Transcription
L	74	3.89	74	3.89	74	3.89	Replication, recombination and repair
B	0	0	0	0	0	0	Chromatin structure and dynamics
D	16	0.84	16	0.84	16	0.84	Cell cycle control, Cell division, chromosome partitioning
V	17	0.84	17	0.89	17	0.84	Defense mechanisms
T	16	0.84	16	0.84	16	0.84	Signal transduction Mechanisms
M	116	6.10	116	6.10	115	6.05	Cell wall/membrane biogenesis
N	10	0.53	10	0.53	10	0.53	Cell motility
U	36	1.89	36	1.89	36	1.89	Intracellular trafficking and secretion
O	68	3.58	68	3.57	68	3.58	Posttranslational modification, protein turnover, chaperones
C	94	4.94	94	4.94	94	4.94	Energy production and conversion
G	85	4.47	85	4.47	87	4.58	Carbohydrate transport and metabolism
E	182	9.57	182	9.56	184	9.68	Amino acid transport and metabolism
F	57	3.00	57	3.00	57	3.00	Nucleotide transport and metabolism
H	80	4.21	80	4.20	80	4.21	Coenzyme transport and metabolism
I	73	3.84	73	3.84	73	3.84	Lipid transport and metabolism
P	74	3.89	74	3.89	76	4.00	Inorganic ion transport and metabolism
Q	40	2.10	40	2.10	40	2.10	Secondary metabolites biosynthesis, transport and catabolism
R	173	9.10	173	9.09	174	9.15	General function prediction only
S	99	5.21	99	5.20	98	5.16	Function unknown
-	574	30.19	576	30.27	575	30.24	Not in COGs

^a^The percentage is based on the total number of protein coding genes in the annotated genome

^b^The total does not correspond to the final quantity of CDSs for each genome because some genes are associated with more than one COG functional category

## Insights from the genome sequence

**Fig. 4:**
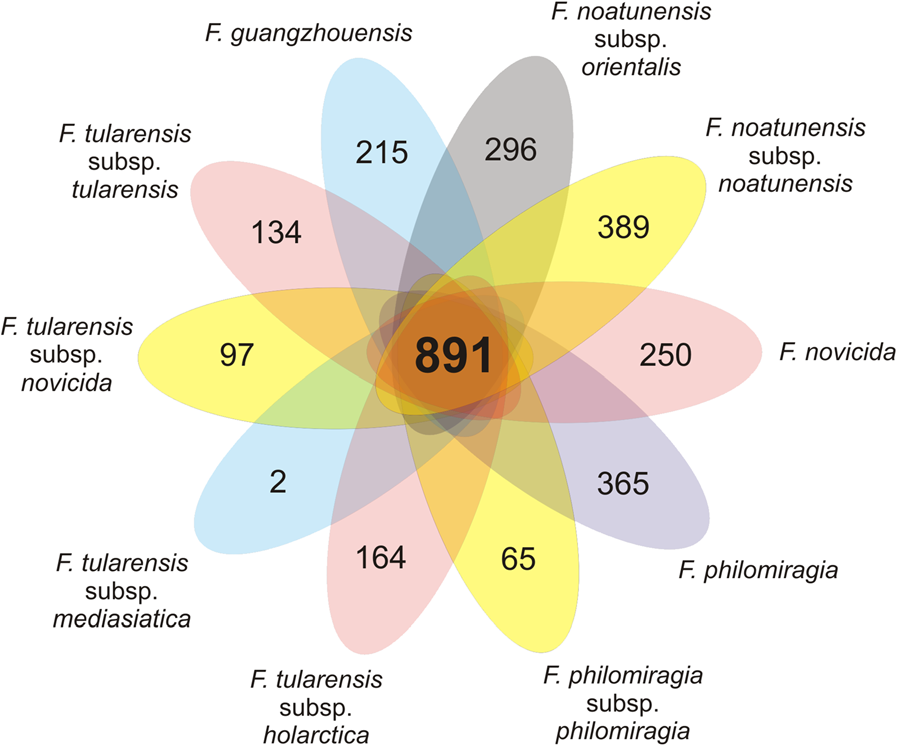
Schematic view of the core genes and singletons of all *Francisella* species in orthoMCL analysis. The central number represents the core CDSs shared by all species, whereas the number on each branch shows the singletons of each species

A high similarity in the genetic content of these genomes was seen in Fig. 3. Additionally, Additional file 1 shows the only eight protein coding sequences with less than 99 % identity between the three sequenced genomes (six hypothetical proteins, one Type IV pili, and one secreted protein). Also, this high intraspecies similarity (100.00 ± 0 %) may be viewed in Additional file 2 and Additional file 3 using Gegenees [20] with threshold of 30 % and Mauve [21] with progessiveMauve algorithm, respectively. These analyses include the three strains of this work and other three deposited at GenBank (FNO01, Toba04, and LADL--07-285A, GenBank nos. CP012153, CP003402, and CP006875, respectively) belonging to the same species. In contrast, the similarity with the subspecies *F. noatunensis* subsp. *noatunensis* is reduced to 84.09 ± 0.40 % (Additional file 2). Furthermore, the orthoMCL software [22] was used to predict the cluster of orthologous genes. CDSs shared by all species were considered to be part of the core genome, whereas CDSs harbored by only species were considered to be species-specific genes. There are 891 CDSs shared by all *Francisella* species (Fig. 4). Interestingly, the *F. tularensis* subsp. *mediasiatica* shows only 2 singleton CDSs, that because this species shared 1380 of yours 1385 CDSs with *F. tularensis* subsp. *tularensis*, whereas the *F. noatunensis* subsp. *orientalis* had 296 species-specific CDSs (Additional file 4 shows COG functional categories found of each CDS). Finally, the GIPSy software [23] was used to predict genomic islands present on *F. noatunensis* subsp. *orientalis*. FNO12 strain was chosen as query, whereas three strains of close related species was used as references (*F. philomiragia* subsp. *philomiragia*ATCC 25017, *F. tularensis* subsp. *novicida* U112, and *Thiomicrospira crunogena* XCL-2, GenBank nos. CP000937, CP000439, CP000109, respectively). Ten genomics islands were predicted by GIPSy, including 2 putative pathogenic islands (PAI1 and PAI2) and 1 putative resistance island (REI1), and plotted using BRIG software [24] (Additional file 5). GEI3 is, apparently, exclusive of *F. noatunensis* subsp. *orientalis**,* and GEI4 is shared only with *F. noatunensis* subsp. *noatunesis* species, another species of marine environment. REI1 and PAI1 are partially shared by all species of *Francisella* genus. PAI2 is partially shared with all species of *Francisella* genus and totally shared with *F. philomiragia* and *F. philomiragia* subsp. *philomiragia* species. GEI6, predicted only as genomic island by GIPSy, contains the genes *mltA, rplM, rpsI, mglA, mglB, rnhB, yfhQ, ptsN, mnmE, cysK, pdpA, pdpB, iglD, iglC, iglB, iglA, pdpD, anmK,* related with the *Francisella* Pathogenicity Island, a previously described pathogenic island for the *Francisella* genus [25]. Further studies are required to characterize these genomic islands, since the GIPSy analysis suggests a greater number of Horizontal Gene Transfer than previously described for this species.

## Conclusions

Three genomes of an important fish pathogen are presented in this work. Despite being isolated from different outbreaks and from different host organs, they are very similar considering the brief analysis of this work. All analyses suggest the clonality of the strains with minor differences in the quantity of pseudogenes and the number of CDSs and RNAs. Furthermore, the high number of pseudogenes present in all sequenced strains corroborate that this species is undergoing genome decay [1].

## Additional files

Additional file 1: Alignment of proteins coding sequences with less than 99 % identity between the three sequenced genomes. (TXT 20 kb) Additional file 2: Heat map showing high similarity between the sequenced genemes performed in Gegenees software with threshold of 30 %. (TIF 984 kb) Additional file 3: Synteny analysis of *Francisella noatunensis* subsp. *orientalis *FNO01, FNO12, FNO24, FNO190, Toba04 and LADL--07-285A strains performed with Mauve software with progessiveMauve algorithm. (TIF 381 kb) Additional file 4: COG functional categories found of each species-specific CDS of *Francisella noatunensis* subsp. *orientalis*. (TXT 16 kb) Additional file 5: The genomic islands predicted by GIPSy software (2 putative pathogenic islands, 1 putative resistance island, and 7 uncharacterized genomic island), plotted using BRIG software. (TIF 2066 kb)

## Abbreviations

CDS: coding sequence
CHAH: cysteine heart agar supplemented with hemoglobin
PGM: personal genome machine
rRNA: ribosomal RNA
tRNA: transporter RNA
