# Supplementary figures and images for: Complete genome sequences of Francisella noatunensis subsp. orientalis strains FNO12, FNO24 and FNO190: a fish pathogen with genomic clonal behavior
Source: Stand Genomic Sci. 2016 Apr 12;11:30. doi: 10.1186/s40793-016-0151-0 (PMC4828924; doi:10.1186/s40793-016-0151-0)

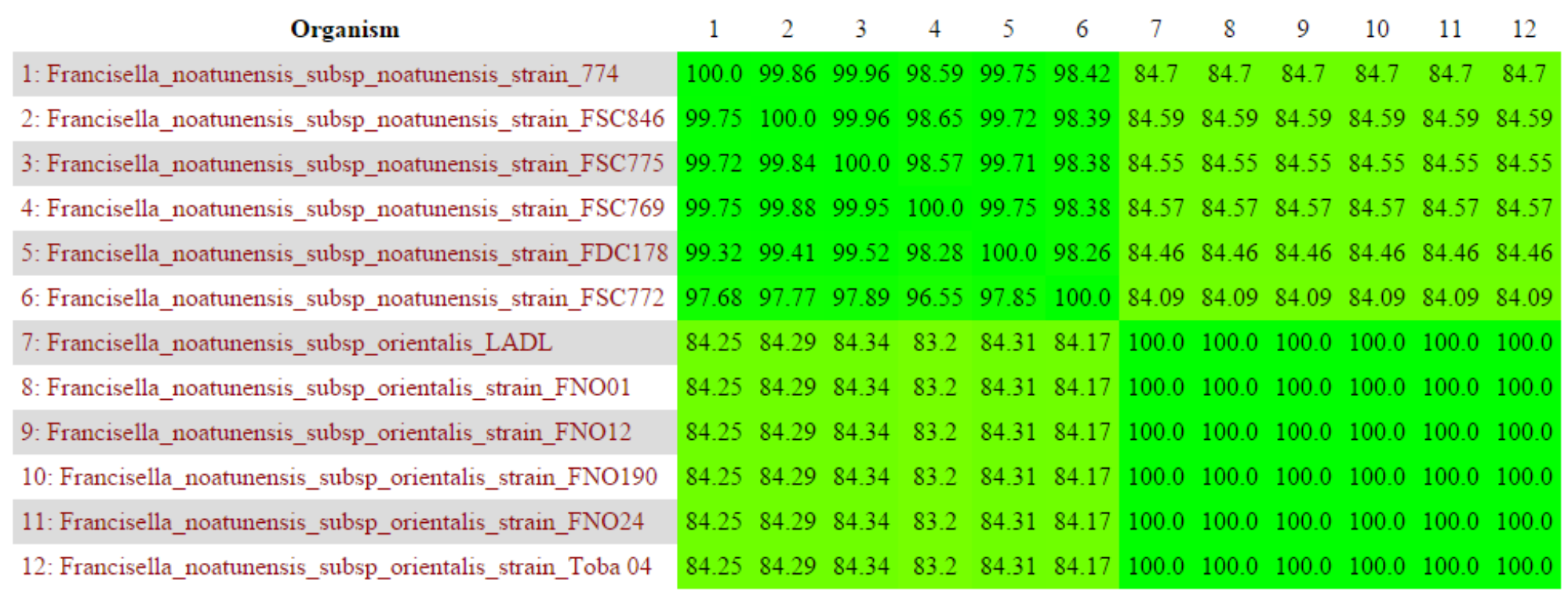

Supplement: Additional file 2: — Heat map showing high similarity between the sequenced genemes performed in Gegenees software with threshold of 30 %. (TIF 984 kb) [file 40793_2016_151_MOESM2_ESM.tif]

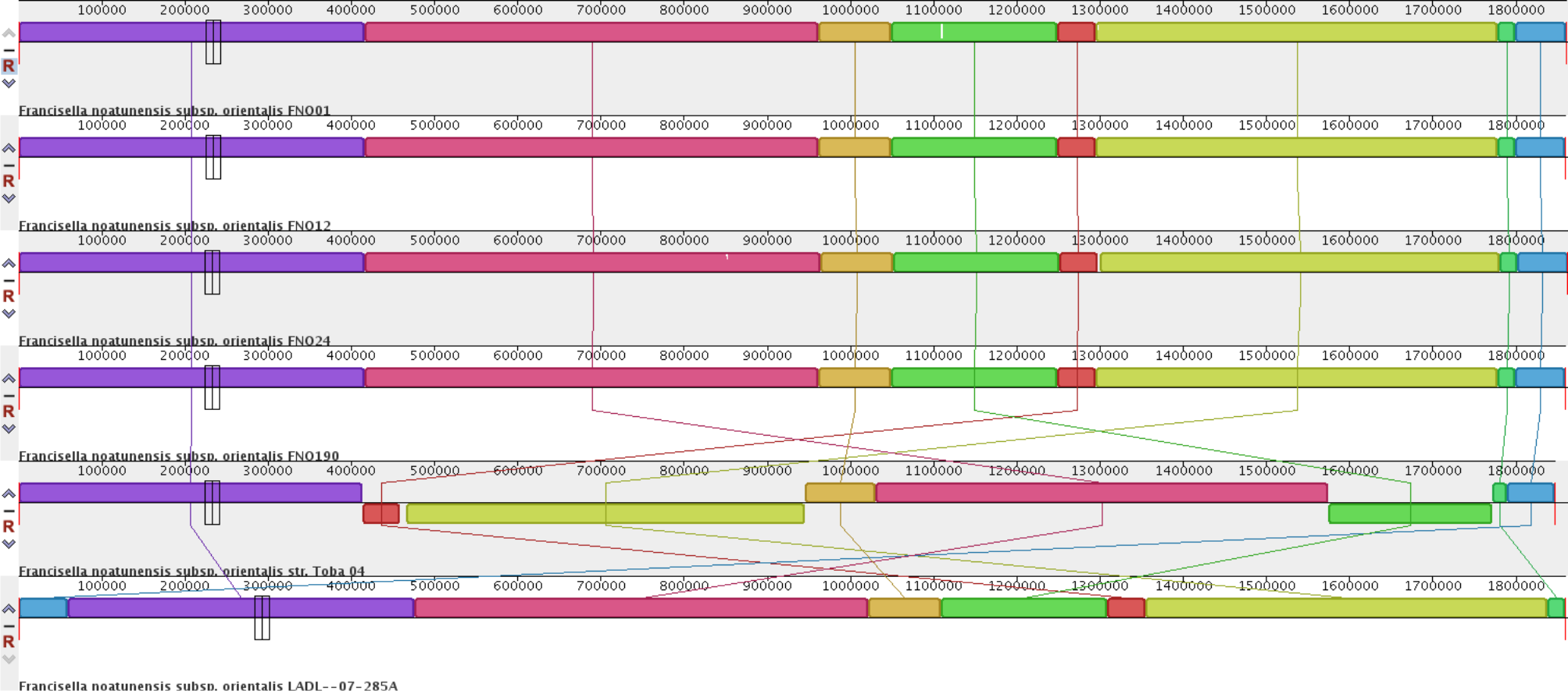

Supplement: Additional file 3: — Synteny analysis of Francisella noatunensis subsp. orientalis FNO01, FNO12, FNO24, FNO190, Toba04 and LADL--07-285A strains performed with Mauve software with progessiveMauve algorithm. (TIF 381 kb) [file 40793_2016_151_MOESM3_ESM.tif]

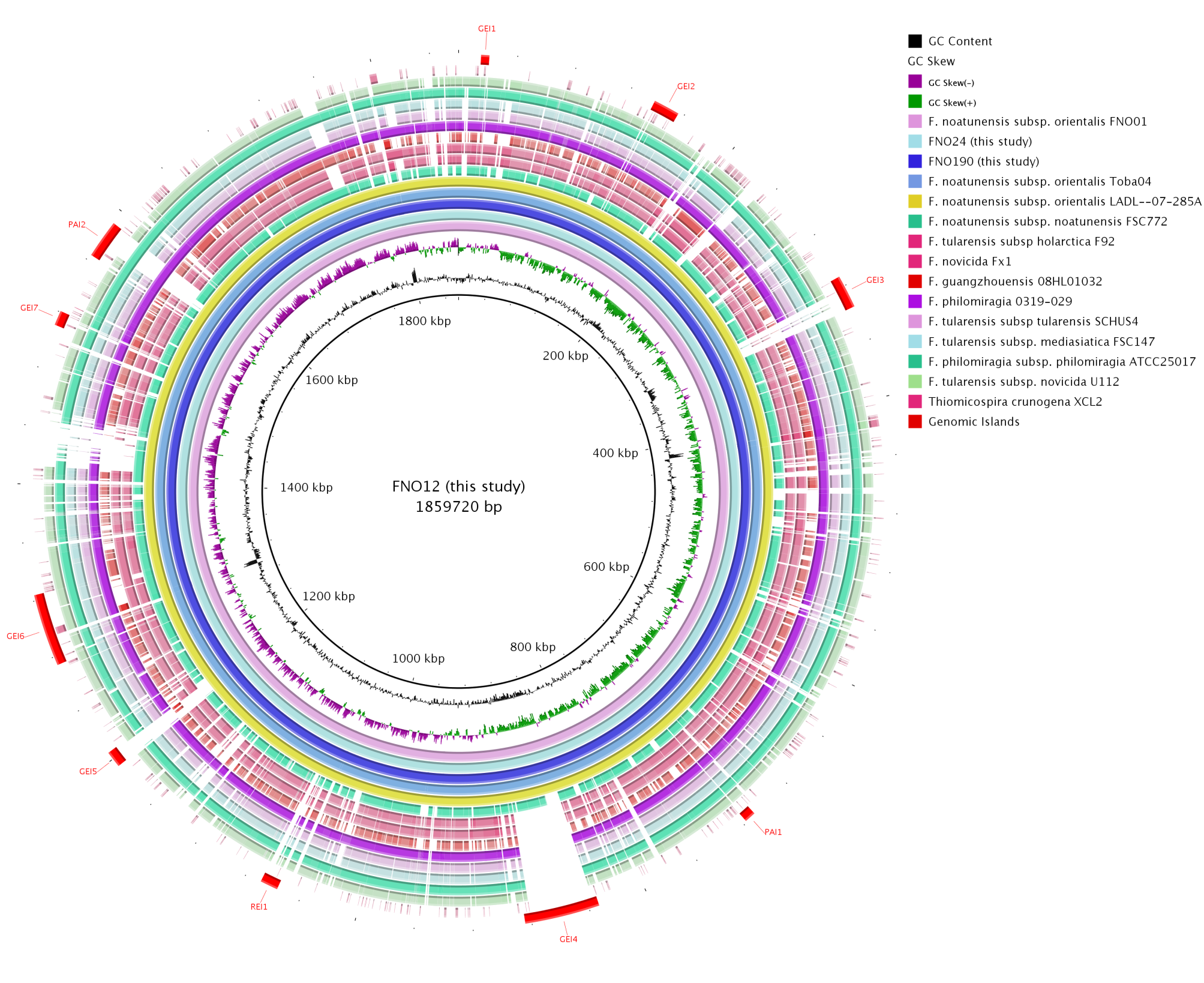

Supplement: Additional file 5: — The genomic islands predicted by GIPSy software (2 putative pathogenic islands, 1 putative resistance island, and 7 uncharacterized genomic island), plotted using BRIG software. (TIF 2066 kb) [file 40793_2016_151_MOESM5_ESM.tif]
